# Towards a unified perinatal theory: Reconciling the births‐based and fetus‐at‐risk models of perinatal mortality

**DOI:** 10.1111/ppe.12537

**Published:** 2019-01-22

**Authors:** K.S. Joseph

**Affiliations:** ^1^ Department of Obstetrics and Gynaecology, School of Population and Public Health University of British Columbia and the Children’s and Women’s Hospital and Health Centre of British Columbia Vancouver British Columbia Canada

**Keywords:** birth, births‐based, fetuses‐at‐risk, gestational age, models, perinatal mortality

## Abstract

**Background:**

There is a need to reconcile the opposing perspectives of the births‐based and fetuses‐at‐risk models of perinatal mortality and to formulate a coherent and unified perinatal theory.

**Methods:**

Information on births in the United States from 2004 to 2015 was used to calculate gestational age‐specific perinatal death rates for low‐ and high‐risk cohorts. Cubic splines were fitted to the fetuses‐at‐risk birth and perinatal death rates, and first and second derivatives were estimated. Births‐based perinatal death rates, and fetuses‐at‐risk birth and perinatal death rates and their derivatives, were examined to identify potential inter‐relationships.

**Results:**

The rate of change in the birth rate dictated the pattern of births‐based perinatal death rates in a triphasic manner: increases in the first derivative of the birth rate at early gestation corresponded with exponential declines in perinatal death rates, the peak in the first derivative presaged the nadir in perinatal death rates, and late gestation declines in the first derivative coincided with an upturn in perinatal death rates. Late gestation increases in the first derivative of the fetuses‐at‐risk perinatal death rate matched the upturn in births‐based perinatal death rates. Differences in birth rate acceleration/deceleration among low‐ and high‐risk cohorts resulted in intersecting perinatal mortality curves.

**Conclusion:**

The first derivative of the birth rate links a cohort's fetuses‐at‐risk perinatal death rate to its births‐based perinatal death rate, and cohort‐specific differences in birth rate acceleration/deceleration are responsible for the intersecting perinatal mortality curves paradox. This mechanistic explanation unifies extant models of perinatal mortality and provides diverse insights.

## 1 INTRODUCTION

Although advances in obstetrics and neonatology have led to substantial reductions in perinatal mortality over the past several decades, a consensus on theoretical issues remains elusive. Extant models of perinatal death include the births‐based formulation (with gestational age‐specific perinatal death rates expressed per 1000 total births at any gestational week) and the extended fetuses‐at‐risk formulation (a survival analysis model from the fetal standpoint, with gestational age‐specific perinatal death rates expressed per 1000 fetuses at risk at any gestational week).^1^, ^2^, ^3^, ^4^, ^5^ The two approaches result in starkly different perspectives: the births‐based model shows that perinatal death rates decline exponentially with increasing gestation, with an upturn at late gestation, while the fetuses‐at‐risk model shows that perinatal death rates increase gradually with increasing pregnancy duration.^1^, ^2^, ^3^, ^4^, ^5^

Perinatology is also plagued by various enigmatic phenomena which have defied easy resolution. Perhaps the most serious challenge to perinatal theory is offered by the paradox of intersecting perinatal mortality curves.^6^, ^7^, ^8^, ^9^, ^10^, ^11^, ^12^, ^13^, ^14^, ^15^, ^16^, ^17^, ^18^ This phenomenon was first described over 50 years ago by Yerushalmy,^6^ who showed that low birthweight infants of women who smoked in pregnancy had lower neonatal mortality (compared with low birthweight infants of non‐smoking women) and the reversal of this mortality difference at higher birthweights. In fact, this mortality crossover is a general phenomenon^18^ seen across many contrasts (eg singletons versus twins, infants of women with versus without hypertension^6^, ^7^, ^8^, ^9^, ^10^, ^11^, ^12^, ^13^, ^14^, ^15^, ^16^, ^17^, ^18^), different outcomes (eg stillbirth, neonatal death,^6^, ^7^, ^8^, ^9^, ^10^, ^11^, ^12^, ^13^, ^14^, ^15^, ^16^, ^17^, ^18^ sudden infant death syndrome^19^, ^20^ and cerebral palsy^21^) and irrespective of how maturity is defined (viz., birthweight or gestational age^6^, ^7^, ^8^, ^9^, ^10^, ^11^, ^12^, ^13^, ^14^, ^15^, ^16^, ^17^, ^18^).

This paper attempts to reconcile the opposing births‐based and fetuses‐at‐risk perspectives of perinatal death and to provide insight into the paradox of intersecting perinatal mortality curves and other perinatal phenomena.

## 2 METHODS

### 2.1 Study rationale

The study was premised on two propositions. The first proposition assumes that the extended fetuses‐at‐risk formulation subsumes the births‐based model because the former deals with fetal and infant processes and events through a longitudinal framework, while the latter addresses these same processes and events at the cross‐sectional moment of birth. The temporal dichotomy between the two models arises because the fetuses‐at‐risk model treats gestational age as survival time, whereas in the births‐based model, gestational age at birth represents a fetal/infant characteristic.^1^, ^2^, ^3^, ^4^, ^5^ It may, therefore, be possible to reformulate the cross‐sectional, births‐based model in terms of the longitudinal, fetuses‐at‐risk model. Note: The time between birth and neonatal death is treated as a latent period and disregarded in both the fetuses‐at‐risk and births‐based calculations of gestational age‐specific neonatal mortality.^5^, ^18^

The second proposition is based on the observation that the exponential increase in gestational age‐specific (extended fetuses‐at‐risk) birth rates complements the exponential decline in births‐based gestational age‐specific perinatal death rates.^5^, ^18^ This commonality (of exponential change) suggests that gestational age‐specific birth rates could represent an explanatory link between the fetuses‐at‐risk gestational age‐specific perinatal mortality rate and the births‐based gestational age‐specific perinatal mortality rate. Specifically, the *rate of change in the birth rate* (ie the first derivative of the birth rate) could be responsible for transforming the gradually rising fetuses‐at‐risk perinatal mortality rate into an exponentially decreasing births‐based perinatal death rate in early gestation. Note: It may be helpful to view the birth rate (births per 1,000 fetus‐weeks) and its first derivative (births per 1000 fetus‐weeks per week, or births per 1000 fetus‐weeks^2^) as being analogous to velocity (metres/sec) and acceleration/deceleration (metres per second per second, or metres per second^2^), respectively. A continuously increasing birth rate (velocity) may conceal large changes in the first derivative of the birth rate (acceleration/deceleration). Changes in the first derivative of the birth rate (analogous to changes in acceleration/deceleration) will have an immediate effect on the birth rate (analogous to velocity) and on the number of births (analogous to distance travelled) that occur in unit time.

### 2.2 Data source and analysis

The study was based on all livebirths and stillbirths in the United States from 2004 to 2015 with data obtained from the fetal death and period‐linked livebirth‐infant death files of the National Center for Health Statistics. The fetal death file only included spontaneous stillbirths (ie pregnancy terminations were not included), gestational age information on all births was based on the clinical estimate of gestation, and births between 20 and 43 weeks were included in the study population. The primary analysis was focused on several cohorts (viz., low‐risk singletons ie singletons of women who did not have hypertension or diabetes mellitus; singletons of women with hypertension; twins; and triplets) in order to demonstrate general applicability. However, for purposes of simplicity, the results presented were restricted to low‐risk singletons and singletons of hypertensive women, with findings from the other two cohorts provided in the Appendix S1.

Gestational age‐specific perinatal death (including stillbirths and neonatal deaths) rates were calculated for each cohort under the births‐based and extended fetuses‐at‐risk formulations. Rates calculated under the fetuses‐at‐risk model were incidence density rates expressed per 1000 fetus‐weeks.^2^, ^18^, ^22^ Although the analyses presented focus on perinatal death rates, rates were also estimated for stillbirths and neonatal deaths separately.^23^

Gestational age‐specific birth rates (ie incidence rates of birth) were calculated using the extended fetuses‐at‐risk formulation.^5^, ^24^ The first and second derivatives of these birth rates and the first and second derivatives of the fetuses‐at‐risk gestational age‐specific perinatal death rates were estimated using the EXPAND procedure in the SAS software package (SAS Institute, Cary, NC). This computation involved fitting a cubic spline to the fetuses‐at‐risk gestational age‐specific birth (or perinatal death) rate and estimating the approximate first derivative of the spline at each gestational week. The second derivative was similarly estimated from a cubic spline fitted to the first derivative. The first derivative of the fetuses‐at‐risk gestational age‐specific birth (or perinatal death) rate quantified the *rate of change *(increase or decrease) in the rate at each gestational week, while the second derivative quantified the *rate of change* in the first derivative. Gestational age‐specific birth rates and the first derivative of the gestational age‐specific birth rates were graphed along with births‐based gestational age‐specific perinatal death rates in order to examine potential relationships at early and late gestation. Similarly, fetuses‐at‐risk gestational age‐specific perinatal death rates and their first derivatives were graphed along with births‐based gestational age‐specific perinatal death rates in order to assess potential associations between these indices.

In supplementary analyses carried out to further address generalisability, other cohorts were examined including singletons of women with diabetes; hypertension and diabetes; older women (≥35 years); younger women (25‐29 years); White women; Black women; and women with and without a previous preterm birth. Analyses were based on anonymised, publicly available data, and ethics approval for the study was not sought.

## 3 RESULTS

The study population included 47 626 172 livebirths and stillbirths between 20 and 43 weeks’ gestation. Perinatal death rates were 8.2 and 13.2 per 1000 total births among low‐risk singletons and singletons of women with hypertension, respectively.

### 3.1 Births‐based and fetuses‐at‐risk perinatal mortality

**Figure 1:**
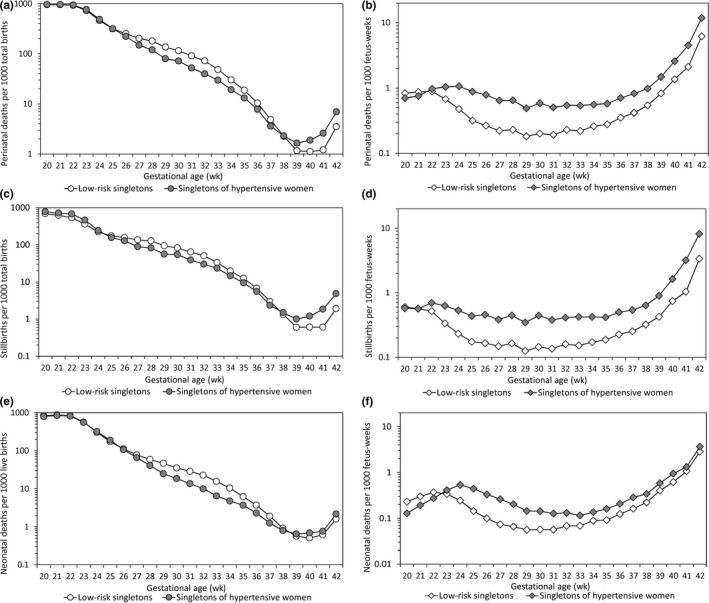
Gestational age‐specific perinatal death, stillbirth and neonatal death rates under the births‐based model (ie per 1000 total births at each gestational week; Panels A, C and E) and under the fetuses‐at‐risk model (ie per 1000 fetus‐weeks; Panels B, D and F) among low‐risk singletons and singletons of women with hypertension, United States, 2004 to 2015

Perinatal mortality rates declined exponentially with increasing gestation until late gestation and then increased exponentially under the births‐based model (Figure 1A). The paradoxical perinatal mortality crossover was evident in the births‐based contrasts: whereas singletons of hypertensive women had lower death rates than low‐risk singletons at early gestation, these differences reversed at late gestation. Under the fetuses‐at‐risk model, rates of perinatal death increased gradually without a crossover (Figure 1B): singletons of hypertensive women had higher mortality at all gestational ages compared with low‐risk singletons. Gestational age‐specific rates of stillbirth and neonatal death showed similar patterns (Figure 1C‐F).

### 3.2 Derivatives of the birth rate and births‐based perinatal mortality at early gestation

**Figure 2:**
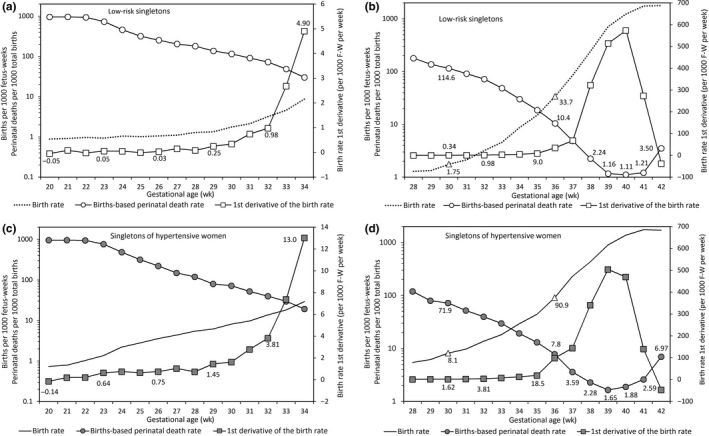
Gestational age‐specific fetuses‐at‐risk birth rates (primary y‐axis), first derivative of the birth rate (secondary y‐axis) and births‐based gestational age‐specific perinatal death rates (primary y‐axis) among low‐risk singletons (Panels A and B) and singletons of women with hypertension (Panels C and D), United States, 2004 to 2015 (gestational age range 20 to 34 weeks in Panels A and C and 28 to 42 weeks in Panels B and D)

Progressive increases in the first derivative of the birth rate at early gestation corresponded with exponential increases in the birth rate and exponential declines in births‐based perinatal death rates (Figure 2, Table 1 and Appendix Table S1)**. **The first derivative of the birth rate increased more substantially, and births‐based perinatal death rates declined to a greater extent at early gestation among singletons of hypertensive women compared with singletons of low‐risk women (Figure 2 and Appendix Table S2). Table 1 also shows the effect of holding the first derivative of the birth rate constant between 29 and 35 weeks’ gestation: birth rates continued to increase and births‐based perinatal death rates continued to decrease but at considerably diminished rates.

**Table 1:** Numbers and rates of perinatal death calculated under the fetuses‐at‐risk and births‐based formulations, low‐risk singletons, United States, 2004 to 2015

Gestational age	Total births	Perinatal deaths	Fetuses‐at‐risk				Births‐based	Assuming a constant first derivative of the birth rate^b^			
			Fetus‐weeks at risk	Birth rate	Birth rate first derivative^a^	Perinatal death rate/1000	Perinatal death rate/1000	First derivative	Birth rate	Total births	Births‐based perinatal death rate/1000
20	36 180	34 300	41 183 376	0.88	−0.05	0.83	948.0				
21	37 520	35 772	41 146 526	0.91	0.08	0.87	953.4				
22	39 463	36 533	41 108 035	0.96	−0.02	0.89	925.8				
23	38 265	27 731	41 069 171	0.93	0.05	0.68	724.7				
24	42 724	19 567	41 028 676	1.04	0.05	0.48	458.0				
25	41 457	13 038	40 986 586	1.01	−0.01	0.32	314.5				
26	43 124	10 863	40 944 295	1.05	0.03	0.27	251.9				
27	44 944	9087	40 900 261	1.10	0.15	0.22	202.2				
28	52 454	9390	40 851 562	1.28	0.08	0.23	179.0				
29	54 409	7404	40 798 131	1.33	0.25	0.18	136.1	0.25	1.33		
30	71 468	8190	40 735 192	1.75	0.34	0.20	114.6	0.25	1.58	64 565	126.9
31	86 129	7811	40 656 394	2.12	0.74	0.19	90.7	0.25	1.83	74 640	104.7
32	129 004	9273	40 548 827	3.18	0.98	0.23	71.9	0.25	2.08	84 661	109.5
33	184 318	8914	40 3921 66	4.56	2.68	0.22	48.4	0.25	2.33	94 621	94.2
34	349 561	10 458	40 125 227	8.71	4.90	0.26	29.9	0.25	2.58	104 514	100.1
35	594 459	11 034	39 653 217	15.0	9.00	0.28	18.6	0.25	2.83	114 330	96.5
36	1 306 146	13 584	38 702 914	33.8	34.2	0.35	10.4		3.08	124 065	109.5
37	3 141 439	15 183	36 479 122	86.1	67.5	0.42	4.83	67.5	86.1		
38	7 555 000	16 905	31 130 902	242.7	322.7	0.54	2.24	67.5	153.6	5 341 807	3.16
39	14 334 153	16 670	20 1863 26	710.1	513.8	0.83	1.16	67.5	221.1	6 507 876	2.56
40	9 897 310	11 002	8 070 594	1226.3	573.1	1.36	1.11	67.5	288.6	6 616 254	1.66
41	2 901 886	3514	1 670 996	1736.6	273.5	2.10	1.21	67.5	356.1	5 807 474	0.61
42	206 584	724	116 761	1769.3	−38.2	6.20	3.50		423.6	4 448 112	0.16
43	13 469	126	13 469	1000.0	‐	9.35	9.35				

a First derivative of the birth rate estimated from the spline fitted to the birth rate (and not the observed birth rate).

b The first derivative of the birth rate was assumed to be constant at 0.25 births per 1000 fetus‐weeks^2^ between 29 and 35 weeks and at 67.5 births per 1000 fetus‐weeks^2^ between 37 and 41 weeks. Total births at each week were calculated based on the fetuses‐at‐risk model and the birth rate (with fetuses at risk at the beginning of each week determined by the number of total births in the previous week).

### 3.3 Peak in the first derivative of the birth rate

Among low‐risk singletons, the first derivative of the birth rate peaked at 40 weeks and the nadir (lowest point) of the births‐based perinatal death rate also occurred at this gestation (Figure 2B). The first derivative of the birth rate peaked at 39 weeks among singletons of women with hypertension (Figure 2D), and the nadir of the births‐based perinatal death rate occurred at 39 weeks as well. The lowest value of the births‐based gestational age‐specific perinatal death rate was higher among the singletons of hypertensive women compared with low‐risk singletons (1.65 vs 1.11 per 1000 total births).

### 3.4 First derivative of the birth rate and births‐based perinatal mortality at late gestation

The first derivative of the birth rate declined sharply at late gestation among low‐risk singletons and singletons of women with hypertension, and this corresponded with an upturn in the births‐based perinatal death rates (Figure 2). Table 1 shows births‐based perinatal mortality rates among low‐risk singletons calculated under the assumption that the first derivative of the birth rate was unchanged between 37 and 41 weeks’ gestation. The late gestation exponential increase in births‐based perinatal mortality rates was abolished without the sharp decrease in the first derivative of the birth rate observed in late gestation.

### 3.5 First derivative of fetuses‐at‐risk perinatal death rate and births‐based death rates

**Figure 3:**
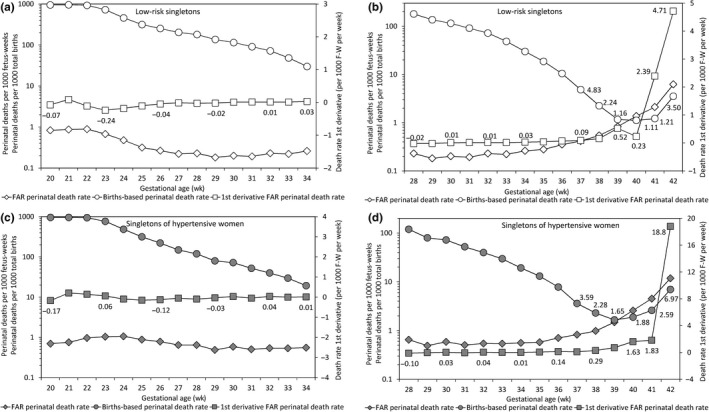
Gestational age‐specific perinatal death rates based on the fetuses‐at‐risk (FAR) model (primary y‐axis), first derivative of the fetuses‐at‐risk perinatal death rate (secondary y‐axis) and births‐based gestational age‐specific perinatal death rates (primary y‐axis) among low‐risk singletons (Panels A and B) and singletons of women with hypertension (Panels C and D), United States, 2004 to 2015 (gestational age range 20 to 34 weeks in Panels A and C and 28 to 42 weeks in Panels B and D)

The first derivative of the fetuses‐at‐risk perinatal death rate was small at early gestation and increased abruptly and substantially late in gestation (Figure 3). This corresponded with the late gestation rise in the births‐based perinatal death rate. The increase in this first derivative was larger among singletons of hypertensive women than among low‐risk singletons (Figure 3).

### 3.6 Derivatives of fetuses‐at‐risk rates and births‐based stillbirth and neonatal death rates

The relationship between the first derivative of the birth rate and the births‐based perinatal death rate, and the relationship between the first derivative of the fetuses‐at‐risk perinatal death rate and the births‐based perinatal death rate were similar to the same relationships between these first derivatives and births‐based stillbirth rates and births‐based neonatal death rates (Appendix Figures S1 and S2).

### 3.7 Birth rate first derivatives and the paradox of intersecting perinatal mortality curves

**Figure 4:**
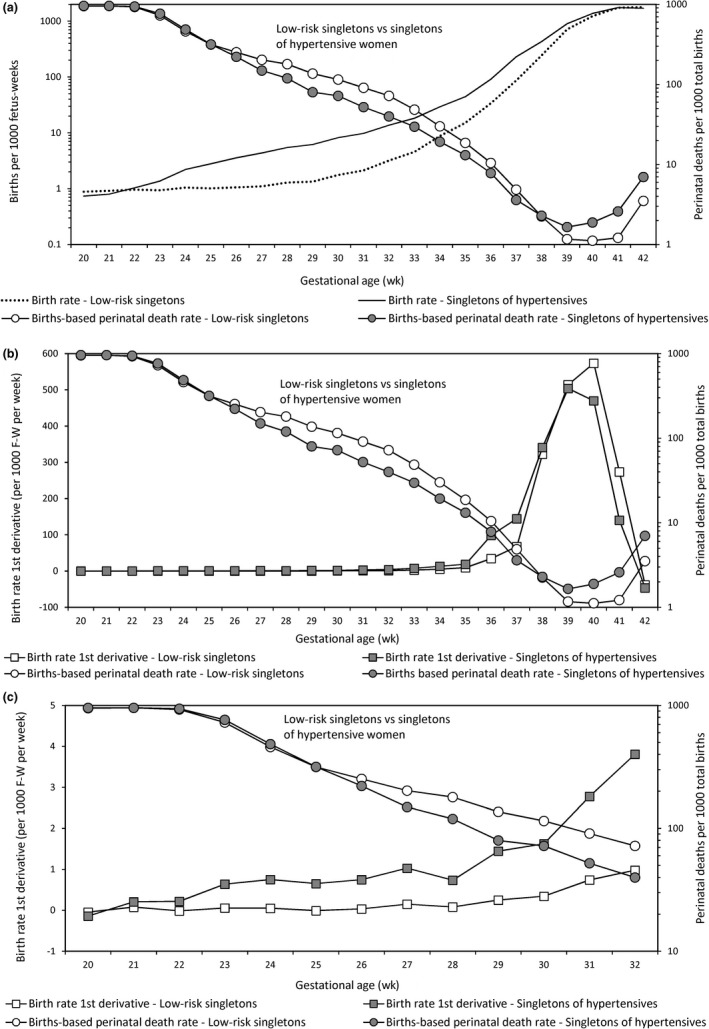
Gestational age‐specific fetuses‐at‐risk birth rates (primary y‐axis; Panel A), first derivative of the birth rate from 20 to 42 weeks (primary y‐axis; Panel B), first derivative of the birth rate from 20 to 32 weeks (primary y‐axis; Panel C) and births‐based perinatal death rates (secondary y‐axis; Panel A, B and C) among low‐risk singletons and singletons of women with hypertension, United States, 2004 to 2015

Larger increases in the first derivative of the birth rate at early gestation among singletons of hypertensive women (vs low‐risk singletons) corresponded with greater declines in births‐based perinatal death rates (Figure 4). The gestational age at which the peak in the first derivative of the birth rate occurred determined the gestational age at which the births‐based perinatal death rate achieved its nadir (Figure 4B). The left shift in the pattern of the first derivative of the birth rate among singletons of hypertensive women (relative to low‐risk singletons) complemented the left‐shift in the births‐based gestational age‐specific perinatal death rate.

### 3.8 Other cohorts

Analyses of twins and triplets showed patterns similar to those described above (Appendix Figures [Link], [Link], [Link], [Link], [Link] and Appendix Tables S3 and S4). Analyses of other cohorts also showed essentially similar patterns (not shown).

## 4 COMMENT

### 4.1 Principal findings

This study shows that the first derivative of the birth rate of a pregnancy cohort follows a triphasic pattern: there is a progressive increase in the first derivative of the birth rate from early gestation onwards, these increases peak, and reverse at late gestation. In other words, there is a progressively increasing acceleration in the birth rate from early gestation onwards, these increases cease, and the pace of acceleration falls sharply to the point of deceleration. In contrast, the fetuses‐at‐risk perinatal death rate shows no change or a modest change in its first derivative at early gestation and a sharp increase in the derivative in late gestation. The progressively increasing acceleration in the birth rate and the lack of a corresponding acceleration in the fetuses‐at‐risk perinatal death rate at early gestation result in a sizeable increase in the number of total births and a far smaller change in the number of perinatal deaths at each gestational week. This manifests as an exponential decline in the births‐based gestational age‐specific perinatal death rate at early gestation. The peak in the first derivative of the birth rate corresponds approximately with the nadir in births‐based perinatal death rates, and subsequent declines in the first derivative of the birth rate coupled with the abrupt increase in the first derivative of the fetuses‐at‐risk perinatal death rate at late gestation lead to an exponential rise in births‐based perinatal death rates at late gestation.

The triphasic correspondence between the first derivative of the birth rate and the births‐based perinatal death rate shows that changes in birth rate acceleration/deceleration largely determine the pattern of births‐based gestational age‐specific perinatal mortality. The critical influence of the first derivative of the birth rate is demonstrated by holding it constant: this abolishes the initial exponential decrease and the subsequent exponential increase in births‐based perinatal deaths at early and late gestation, respectively. The triphasic pattern of the first derivative of the birth rate is left shifted in high‐risk cohorts, and this results in a left shift in the triphasic pattern of births‐based gestational age‐specific perinatal mortality rates and the paradox of intersecting perinatal mortality curves. The details and implications of these findings are provided below.

#### 4.1.1 The birth rate, its first derivative and the gestational age distribution

The first derivative of the birth rate determines the subsequent birth rate and the number of births in the time interval (just as acceleration/deceleration determines subsequent velocity and the distance travelled in unit time). The pattern of the first derivative of the birth rate thus determines the gestational age distribution of a cohort, while this pattern and other influences (viz., the growth rate pattern) determine the birthweight distribution.

#### 4.1.2 Fetuses‐at‐risk and births‐based stillbirth and neonatal death rates

The triphasic concordance between the first derivative of the birth rate and births‐based perinatal death rates is also seen in the relationship between the first derivative of the birth rate and births‐based stillbirth rates, and in the relationship between the first derivative of the birth rate and births‐based neonatal death rates. The large, progressive increases in the first derivative of the birth rate and absent or modest changes in the first derivative of the fetuses‐at‐risk stillbirth and neonatal death rates at early gestation lead to substantially increasing numbers of non‐compromised fetuses being delivered at each gestational week along with a gradually changing number of compromised fetuses (that end in stillbirth or which are destined to die in the neonatal period). This manifests as an exponentially declining births‐based gestational age‐specific stillbirth and neonatal death rate at early gestation. The exponential rise in births‐based stillbirth and births‐based neonatal death rates at late gestation is due to a reversal of these phenomena, that is, smaller increases in non‐compromised births (due to a declining acceleration in the birth rate) and larger increases in compromised births (due to a progressive acceleration in the fetuses‐at‐risk stillbirth/neonatal death rate).

The fetuses‐at‐risk perinatal death rate at any gestational week has been previously represented as a product of the fetuses‐at‐risk birth rate and the births‐based perinatal death rate.^23^, ^25^, ^26^ In fact, temporal considerations, and the triphasic concordance between the first derivative of the birth rate and the births‐based perinatal death rate, suggest that the births‐based perinatal death rate is preferably conceptualised as a consequence of the fetuses‐at‐risk birth rate and the fetuses‐at‐risk perinatal death rate. This perspective implies that the birth rate, the fetuses‐at‐risk gestational age‐specific stillbirth rate and the fetuses‐at‐risk gestational age‐specific neonatal death rate quantify primary phenomena. On the other hand, the births‐based gestational age‐specific stillbirth rate and the births‐based gestational age‐specific neonatal death rate quantify secondary phenomena that arise at the cross‐sectional moment of birth when birth rates and fetuses‐at‐risk stillbirth/neonatal death rates intersect.

#### 4.1.3 Late gestation upturn in births‐based perinatal mortality

Fetal post‐maturity, the traditional explanation for the late gestation upturn in births‐based perinatal death rates, represents an insufficiently plausible mechanism for the observed exponential rise in mortality at term and post‐term gestation. This study provides an alternative explanation: the exponential increase in births‐based perinatal death rates at late gestation is the consequence of sharp reductions in birth rate acceleration and sharp increases in acceleration of the fetuses‐at‐risk perinatal death rate. The post‐maturity explanation is less compelling because it suggests a fetal (post‐maturity) mechanism for the phenomenon, whereas declines in the first derivative of the birth rate and increases in the first derivative of the fetuses‐at‐risk perinatal death rate imply utero‐placental mechanisms. The latter are more consistent with the evidence from animal and human studies (see below).

#### 4.1.4 Parturition as a hypersensitivity‐type phenomenon

The relatively large, isolated increase in the first derivative of the birth rate and the lack of any substantial change in the fetuses‐at‐risk perinatal death rate at early gestation suggest that parturition is the primary response to adverse influences in pregnancy. The magnitude of the difference in these two first derivatives may also indicate that parturition represents an exaggerated, hypersensitivity‐type reaction to adverse influences. Sensitisation of oxytocin receptors (or similar mechanisms) in high‐risk pregnancy likely causes the myometrium to transition from quiescent to contractile in early gestation. A combination of compromised and non‐compromised fetuses is delivered at early gestation, with the proportion of compromised fetuses born being lower in cohorts manifesting higher birth rates. It is possible that the relatively high rate of parturition at early gestation, which characterises high‐risk cohorts, represents an evolutionary mechanism that prioritises maternal survival in the face of potential threats to fetal well‐being.

#### 4.1.5 Biological basis for birth patterns and late gestation changes in perinatal mortality

Animal and human studies show that the ability of the utero‐placental unit to support the fetus diminishes steadily as gestation advances.^27^, ^28^, ^29^ It is estimated that the average uterine blood flow volume per unit of estimated fetal weight declines from 993 mL/min/kg at 24 weeks to 360 mL/min/kg at 34 weeks and to 296 mL/min/kg at 38 weeks’ gestation in humans.^28^ This provides the theoretical basis for the obstetric practice of routinely inducing labour in all pregnancies at post‐term gestation and even earlier in high‐risk pregnancies.^30^ The progressive decline in utero‐placental blood flow from mid‐gestation onwards is also more consistent with the fetuses‐at‐risk pattern of gestational age‐specific perinatal mortality (which shows increases in death rates well before term gestation, Figure 3B,D) than with the births‐based pattern of gestational age‐specific perinatal mortality (which only shows an upturn in death rates at term or post‐term gestation, Figure 1A).

The incremental decline in utero‐placental blood flow per unit fetal weight from mid‐gestation onwards may also explain the progressive increase in the first derivative of the birth rate, although it is unclear why this first derivative declines sharply at late gestation. One possible explanation is a relative depletion of susceptibles, with pregnancies that reach late gestation being less responsive to hormonal and other triggers that initiate parturition.

#### 4.1.6 Paradox of intersecting perinatal mortality curves

At early gestation, high‐risk populations show substantial increases in the first derivative of the birth rate, high birth rates, relatively modest or no change in fetuses‐at‐risk perinatal death rates, a larger proportion of non‐compromised births and relatively low births‐based perinatal death rates. The peak and subsequent decline in the first derivative of the birth rate among high‐risk cohorts occur earlier in gestation, and hence, the nadir and subsequent increase in the births‐based perinatal death rate in such populations also occur at earlier gestation. Differences in the first derivative of the birth rate between low‐ and high‐risk cohorts (ie a left shift in the pattern of the first derivative of the birth rate in high‐risk cohorts) are thus the key factor responsible for the paradox of intersecting perinatal mortality curves.

#### 4.1.7 Explanations for the paradox in contemporary epidemiology

Proponents of the fetuses‐at‐risk formulation, which eliminates the intersecting perinatal mortality curves paradox, conceptualise the births‐based paradox as being the product of a non‐causal model.^5^, ^18^ Non‐causal models, which serve an important diagnostic or (non‐causal) prognostic purpose, typically make liberal use of interaction and other product terms. The inclusion of interaction (or other terms) in non‐causal models is justified solely by their performance as predictors, and terms in non‐causal models do not require causal interpretation (unlike those in causal models). Thus, serologic tests, widely used to diagnose specific infections, are judged solely on the basis of diagnostic performance, and causal interpretation, specifically reverse causality between immune response and infection, is ignored. Similarly, the births‐based model provides non‐causal prognosis: mothers of small babies who smoked in pregnancy can be reassured that their infants have a relatively better prognosis, with no associated causal implication regarding the effect of maternal smoking on mortality.

Relative birthweight and gestational age formulations also resolve the paradox of intersecting mortality curves.^9^, ^10^, ^11^, ^12^, ^13^, ^14^, ^15^, ^31^ The mechanism by which these formulations eliminate the paradox may be explained using the fetuses‐at‐risk birth rate and the first derivative of the birth rate.^32^

Collider stratification bias,^33^, ^34^, ^35^ another explanation for the paradox, posits that perinatal mortality curves that intersect across any determinant contrast (eg twins vs singletons) are a consequence of stratification on a variable (eg gestational age) that is the common effect of the determinant in question and an unmeasured or unknown confounder (of the gestational age and perinatal death relation).^33^ Although this bias is a well‐recognised phenomenon, the inability to identify plausible candidates, which could serve as the unmeasured confounder in the context of the paradox, potentially weakens this explanation. Candidates proposed as unmeasured confounders include factors which only apply to specific contrasts (eg placental abruption in the pre‐eclampsia and cerebral palsy relation)^35^ or inadequately specified, generic factors (eg genotype or placental proteins).^33^ Perhaps a better contender for this confounder role is the (gestational age‐specific) birth rate since this is a determinant of the stratification variable involved in the paradox (ie gestational age) and also the outcome (perinatal death). However, this “mixed models” explanation is perhaps less appealing than the simpler argument that interaction and other terms in non‐causal prognostic models do not require causal clarification.

#### 4.1.8 Perspectives in obstetrics and neonatology

Until recently, the exponential decline in perinatal death rates with increasing gestation seen under the births‐based perspective precluded the development of an epidemiologic framework for justifying medically indicated early delivery.^4^ Such early delivery affects the pattern of the first derivative of the birth rate; the latter thus represents the population counterpart of medically indicated early delivery in obstetrics. Populations in high‐income countries have likely shown temporal changes in the first derivative of the birth rate in recent decades that correspond with temporal declines in perinatal mortality.

In neonatology, the births‐based perspective has led to potentially flawed international comparisons of neonatal mortality among infants born at very preterm gestation.^36^, ^37^ Truncated contrasts of very preterm populations provide a distorted perspective (from a causal standpoint) because they are confined to the early gestation part of the (births‐based) intersecting perinatal mortality curve. Not surprisingly, studies based on infants <32 weeks’ gestation have paradoxically shown lower neonatal death rates among infants of older mothers compared with the infants of younger mothers,^38^ and lower neonatal death rates among infants of women with hypertension compared with infants of normotensive women.^39^

#### 4.1.9 Utility of the fetuses‐at‐risk and births‐based models

The findings of this study support the contention that the fetuses‐at‐risk and births‐based models are suitable for addressing different epidemiologic issues; the longitudinal, survival analysis perspective of the fetuses‐at‐risk model is preferable for addressing causal questions, while the cross‐sectional births‐based model is excellent for setting prognosis at birth.^5^, ^18^, ^24^ This difference arises because the fetuses‐at‐risk formulation represents a survival analysis model and because critical biological phenomena (including birth rates) can only be represented in this framework. On the other hand, the births‐based approach derives its strength as a prognostic model from its use of gestational age at birth and birthweight as powerful predictors of mortality and other outcomes of perinatal interest. Epidemiologic questions in medicine fall into causal and non‐causal categories, and the fetuses‐at‐risk and births‐based models provide the framework for research in causal and non‐causal perinatal domains, respectively.

#### 4.1.10 Directions for future research

The exaggerated parturition response at early gestation highlights the sensitivity of the uterus to adverse influences and underscores the need for a tocolytic. Effective tocolysis at early gestation, combined with serial fetal monitoring, is likely to result in the continuation of many high‐risk pregnancies involving non‐compromised fetuses that would otherwise be spontaneously delivered at preterm gestation. Also, obstetric practice at late gestation in specific high‐risk cohorts needs to be periodically evaluated by correlating iatrogenic changes in the first derivative of the birth rate with changes in perinatal mortality and neonatal morbidity.

### 4.2 Strengths of the study

This study offers a relatively simple, mechanistic explanation that unifies the fetuses‐at‐risk and births‐based models of gestational age‐specific perinatal mortality and also provides insights into diverse issues.

### 4.3 Limitations of the data

Inaccuracies in gestational age ascertainment may have slightly altered the gestational week at which the first derivative of the birth rate peaked and the births‐based perinatal death rate attained its nadir. Gestational age information in our data source was available in completed weeks, whereas information in days would have provided more precision in fitting splines and estimating derivatives. Calculations of fetuses‐at‐risk incidence density rates assumed that births were uniformly distributed across each gestational week, although this assumption was likely compromised especially remote from 39 weeks’ gestation. The study cohort was likely affected by increasing birth registration at the borderline of viability in recent years, although changes at 20‐24 weeks would not have substantially altered study findings, which were most evident at gestational ages well past viability. Finally, it was not possible to model fetal growth restriction rates as such data were not available, and proxy measures such as revealed growth restriction are influenced by birth rates.^24^

## 5 INTERPRETATION AND CONCLUSIONS

The first derivative of the birth rate in any cohort links the fetuses‐at‐risk perinatal death rate and the births‐based perinatal death rate; its progressive increase in early gestation leads to an exponential decline in births‐based perinatal death rates; and its sharp decline in late gestation results in an exponential increase in births‐based perinatal death rates (though the latter is also affected by a late gestation increase in the first derivative of the fetuses‐at‐risk perinatal death rate). The pattern of the first derivative of the birth rate in high‐risk cohorts is left shifted and this leads to a left shift in the births‐based perinatal death rate, which manifests as the paradox of intersecting perinatal mortality curves. Changes in the acceleration and deceleration in birth rates at early and late gestation thus provide a unifying explanation that links the two seemingly opposed perspectives of the fetuses‐at‐risk and births‐based models of gestational age‐specific perinatal mortality.
