# Supplementary material for: Towards a unified perinatal theory: Reconciling the births‐based and fetus‐at‐risk models of perinatal mortality
Source: Paediatr Perinat Epidemiol. 2019 Jan 22;33(2):101–12. doi: 10.1111/ppe.12537 (PMC6487839; doi:10.1111/ppe.12537)
Supplement: Supplementary file 1 [file PPE-33-101-s001.docx]

**Appendix**

**Appendix 1**

Table of contents Page

Table of contents 1

Table S1. Numbers and rates of live births, and perinatal deaths calculated under the fetuses- 3

at-risk and births-based models, **low-risk** s**ingletons**, United States, 2004 to 2015.

Table S2. Numbers and rates of live births, and perinatal deaths calculated under the fetuses- 4

-at-risk and births-based models, s**ingletons of women with hypertension**,

United States, 2004 to 2015.

Table S3. Numbers and rates of live births, and perinatal deaths calculated under the fetuses 5

-at-risk and births-based models, **twins**, United States, 2004 to 2015.

Table S4. Numbers and rates of live births, and perinatal deaths calculated under the fetuses 6

-at-risk and births-based models, **triplets**, United States, 2004 to 2015.

**Appendix 2**

Figure 1. Gestational age-specific fetuses-at-risk birth rates (primary Y-axis), first 1

derivative of the birth rate (secondary Y-axis), births-based stillbirth rates

(primary Y-axis; Panels A and C) and births-based neonatal death rates

(Panels B and D) among **low-risk singletons** (Panels A and B) and **singletons**

**of women with hypertension** (Panels C and D), United States, 2004 to 2015.

Figure 2. Gestational age-specific stillbirth (primary Y-axis; Panel A and C) and neonatal 2

death rates (primary Y-axis; Panel B and D), based on the fetuses-at-risk (FAR)

model, first derivative of the fetuses-at-risk stillbirth and neonatal death rates

(secondary Y-axis) and births-based gestational age-specific stillbirth and neonatal

death rates (primary Y-axis) among **low-risk singletons** (Panels A and B) and

**singletons of women with hypertension** (Panels C and D), United States, 2004 to

2015.

Figure 3. Gestational age-specific perinatal death, stillbirth and neonatal death rates under 3

the births-based (i.e., per 1,000 total births at each gestational week; Panels A,

C and E) and fetuses-at-risk models (i.e., per 1,000 fetus-weeks; Panels B, D

and F) among **low-risk singletons, singletons of women with hypertension,**

**twins and triplets**, United States, 2004 to 2015.

Figure 4. Gestational age-specific fetuses-at-risk birth rates (primary Y-axis), first 4

derivative of the birth rate (secondary Y-axis) and births-based gestational

age-specific perinatal death rates (primary Y-axis) among **twins** (Panels A

and B) and **triplets** (Panels C and D), United States, 2004 to 2015 (gestational

age range 20 to 34 weeks, 26 to 40 in Panel B and 23 to 37 in Panels D).

Figure 5. Gestational age-specific perinatal death rates based on the fetuses-at-risk 5

(FAR) model (primary Y-axis), first derivative of the fetuses-at-risk perinatal

death rate (secondary Y-axis) and births-based gestational age-specific

perinatal death rates (primary Y-axis) among **twins** (Panels A and B) and

**triplets** (Panel C and D), United States, 2004 to 2015 (gestational age range 20

to 34 weeks in Panels A and C, 26 to 40 weeks in Panel B and 23 to 37 in Panel

D).

Table of contents Page

Figure 6. Gestational age-specific fetuses-at-risk birth rates (primary Y-axis; Panel A), first 6

derivative of the birth rate from 20 to 42 weeks (primary Y-axis; Panel B), first

derivative of the birth rate from 20 to 32 weeks (primary Y-axis; Panel C), and

births-based perinatal death rates (secondary Y-axis; Panel A, B and C) among

**low-risk singletons and twins**, United States, 2004 to 2015.

Figure 7. Gestational age-specific fetuses-at-risk birth rates (primary Y-axis; Panel A), first 7

derivative of the birth rate from 20 to 42 weeks (primary Y-axis; Panel B), first

derivative of the birth rate from 20 to 32 weeks (primary Y-axis; Panel C), and

births-based perinatal death rates (secondary Y-axis; Panel A, B and C) among

**low-risk singletons and triplets**, United States, 2004 to 2015.

Appendix Table S1. Numbers and Rates of Live births, Stillbirths, Neonatal Deaths and Perinatal Deaths Calculated Under the Fetuses-At-Risk and Births-Based

Formulations, **Low-risk** **Singletons (Without Maternal Hypertension or Diabetes Mellitus)**, United States 2004 to 2015.

| Gestational  age | Total | Still- | Neonatal | Fetuses-at-risk formulation | | | | | | | | |  | Births-based formulation | | |
| --- | --- | --- | --- | --- | --- | --- | --- | --- | --- | --- | --- | --- | --- | --- | --- | --- |
|  | births | births | deaths | Fetus- | Birth | Birth rate | | Still- | Neonatal | Perinatal | Perinatal death rate | |  | Still- | Neonatal | Perinatal |
|  |  |  |  | weeks | rate | derivatives | | birth | death | death | rate derivatives | |  | birth | death | death |
|  |  |  |  | at risk |  | D1 | D2 | rate | rate | rate | D1 | D2 |  | rate | rate | rate |
| 20 | 36,180 | 24,796 | 9,504 | 41,183,376 | 0.88 | -0.05 | 0.42 | 0.60 | 0.23 | 0.83 | -0.07 | 0.50 |  | 685.4 | 834.9 | 948.0 |
| 21 | 37,520 | 23,418 | 12,354 | 41,146,526 | 0.91 | 0.08 | -0.08 | 0.57 | 0.30 | 0.87 | 0.09 | -.10 |  | 624.2 | 876.1 | 953.4 |
| 22 | 39,463 | 21,272 | 15,261 | 41,108,035 | 0.96 | -0.02 | -0.02 | 0.52 | 0.37 | 0.89 | -0.11 | -0.21 |  | 539.0 | 838.9 | 925.8 |
| 23 | 38,265 | 13,777 | 13,954 | 41,069,171 | 0.93 | 0.05 | 0.07 | 0.34 | 0.34 | 0.68 | -0.24 | -0.03 |  | 360.0 | 569.8 | 724.7 |
| 24 | 42,724 | 9,496 | 10,071 | 41,028,676 | 1.04 | 0.05 | -0.06 | 0.23 | 0.25 | 0.48 | -0.18 | 0.09 |  | 222.3 | 303.1 | 458.0 |
| 25 | 41,457 | 7,121 | 5,917 | 40,986,586 | 1.01 | -0.01 | -0.03 | 0.17 | 0.14 | 0.32 | -0.10 | 0.07 |  | 171.8 | 172.3 | 314.5 |
| 26 | 43,124 | 6,772 | 4,091 | 40,944,295 | 1.05 | 0.03 | 0.13 | 0.17 | 0.10 | 0.27 | -0.04 | 0.05 |  | 157.0 | 112.5 | 251.9 |
| 27 | 44,944 | 6,071 | 3,016 | 40,900,261 | 1.10 | 0.15 | -0.01 | 0.15 | 0.07 | 0.22 | -0.01 | 0.01 |  | 135.1 | 77.6 | 202.2 |
| 28 | 52,454 | 6,697 | 2,693 | 40,851,562 | 1.28 | 0.08 | 0.05 | 0.16 | 0.07 | 0.23 | -0.02 | -0.01 |  | 127.7 | 58.9 | 179.0 |
| 29 | 54,409 | 5,115 | 2,289 | 40,798,131 | 1.33 | 0.25 | 0.12 | 0.13 | 0.06 | 0.18 | -0.02 | 0.02 |  | 94.0 | 46.4 | 136.1 |
| 30 | 71,468 | 5,871 | 2,319 | 40,735,192 | 1.75 | 0.34 | 0.27 | 0.14 | 0.06 | 0.20 | 0.01 | 0.02 |  | 82.2 | 35.4 | 114.6 |
| 31 | 86,129 | 5,503 | 2,308 | 40,656,394 | 2.12 | 0.74 | 0.26 | 0.14 | 0.06 | 0.19 | 0.01 | 0.00 |  | 63.9 | 28.6 | 90.7 |
| 32 | 129,004 | 6,484 | 2,789 | 40,548,827 | 3.18 | 0.98 | 0.59 | 0.16 | 0.07 | 0.23 | 0.01 | -0.00 |  | 50.3 | 22.8 | 71.9 |
| 33 | 184,318 | 6,133 | 2,781 | 40,3921,66 | 4.56 | 2.68 | 3.21 | 0.15 | 0.07 | 0.22 | 0.01 | 0.01 |  | 33.3 | 15.6 | 48.4 |
| 34 | 349,561 | 6,895 | 3,563 | 40,125,227 | 8.71 | 4.90 | -1.63 | 0.17 | 0.09 | 0.26 | 0.03 | 0.02 |  | 19.7 | 10.4 | 29.9 |
| 35 | 594,459 | 7,403 | 3,631 | 39,653,217 | 15.0 | 9.00 | 22.3 | 0.19 | 0.09 | 0.28 | 0.04 | 0.02 |  | 12.5 | 6.19 | 18.6 |
| 36 | 1,306,146 | 8,778 | 4,806 | 38,702,914 | 33.8 | 34.2 | 0.44 | 0.23 | 0.12 | 0.35 | 0.07 | 0.04 |  | 6.72 | 3.70 | 10.4 |
| 37 | 3,141,439 | 9,244 | 5,939 | 36,479,122 | 86.1 | 67.5 | 151.3 | 0.25 | 0.16 | 0.42 | 0.09 | -0.04 |  | 2.94 | 1.90 | 4.83 |
| 38 | 7,555,000 | 10,005 | 6,900 | 31,130,902 | 242.7 | 322.7 | 259.3 | 0.32 | 0.22 | 0.54 | 0.16 | 0.39 |  | 1.32 | 0.91 | 2.24 |
| 39 | 14,334,153 | 8,556 | 8,114 | 20,1863,26 | 710.1 | 513.8 | 150.5 | 0.42 | 0.40 | 0.83 | 0.52 | -0.21 |  | 0.60 | 0.57 | 1.16 |
| 40 | 9,897,310 | 5,984 | 5,018 | 8,070,594 | 1226.3 | 573.1 | -109.9 | 0.74 | 0.62 | 1.36 | 0.23 | 0.68 |  | 0.60 | 0.51 | 1.11 |
| 41 | 2,901,886 | 1,736 | 1,778 | 1,670,996 | 1736.6 | 273.5 | -431.8 | 1.04 | 1.06 | 2.10 | 2.39 | 3.12 |  | 0.60 | 0.61 | 1.21 |
| 42 | 206,584 | 393 | 331 | 116,761 | 1769.3 | -38.2 | 3.18 | 3.37 | 2.83 | 6.20 | 4.71 | 0.29 |  | 1.90 | 1.61 | 3.50 |
| 43 | 13,469 | 69 | 57 | 13,469 | 1000.0 | 669.6 | 1607.3 | 5.12 | 4.23 | 9.35 | 0.50 | -9.94 |  | 5.12 | 4.25 | 9.35 |

* Derivatives of the birth rate (D1 and D2) estimated from the splines fitted to the original rate (and not the observed rate).

Appendix Table S2. Numbers and Rates of Live births, Stillbirths, Neonatal Deaths and Perinatal Deaths Calculated Under the Fetuses-At-Risk and Births-Based Formulations, **Singletons with Maternal Hypertension**, United States 2004 to 2015.

| Gestational  age | Total | Still- | Neonatal | Fetuses-at-risk formulation | | | | | | | | |  | Births-based formulation | | |
| --- | --- | --- | --- | --- | --- | --- | --- | --- | --- | --- | --- | --- | --- | --- | --- | --- |
|  | births | births | deaths | Fetus- | Birth | Birth rate | | Still- | Neonatal | Perinatal | Perinatal death | |  | Still- | Neonatal | Perinatal |
|  |  |  |  | weeks | rate | derivatives* | | birth | death | death | rate derivatives | |  | birth | death | death |
|  |  |  |  | at risk |  | D1 | D2 | rate | rate | rate | D1 | D2 |  | rate | rate | rate |
| 20 | 1,673 | 1,305 | 292 | 2,280,759 | 0.73 | -0.14 | 0.91 | 0.57 | 0.13 | 0.70 | -0.17 | 0.78 |  | 780.0 | 793.5 | 954.6 |
| 21 | 1,806 | 1,285 | 440 | 2,279,019 | 0.79 | 0.21 | -0.02 | 0.56 | 0.19 | 0.76 | 0.21 | 0.06 |  | 711.5 | 844.5 | 955.2 |
| 22 | 2,351 | 1,588 | 621 | 2,276,941 | 1.03 | 0.22 | 0.24 | 0.70 | 0.27 | 0.97 | 0.15 | -0.10 |  | 675.5 | 813.9 | 939.6 |
| 23 | 3,103 | 1,426 | 938 | 2,274,214 | 1.36 | 0.64 | 0.35 | 0.63 | 0.41 | 1.04 | 0.06 | -0.13 |  | 459.6 | 559.3 | 761.8 |
| 24 | 5,004 | 1,206 | 1,212 | 2,270,160 | 2.20 | 0.75 | -0.06 | 0.53 | 0.53 | 1.07 | -0.10 | -0.12 |  | 241.0 | 319.1 | 483.2 |
| 25 | 6,322 | 986 | 1,008 | 2,264,497 | 2.79 | 0.65 | -0.07 | 0.44 | 0.45 | 0.88 | -0.15 | -0.01 |  | 156.0 | 188.9 | 315.4 |
| 26 | 8,060 | 1,031 | 749 | 2,257,306 | 3.57 | 0.75 | 0.34 | 0.46 | 0.33 | 0.79 | -0.13 | 0.08 |  | 127.9 | 106.6 | 220.8 |
| 27 | 9,774 | 865 | 589 | 2,248,389 | 4.35 | 1.03 | -0.15 | 0.38 | 0.26 | 0.65 | -0.05 | 0.01 |  | 88.5 | 66.1 | 148.8 |
| 28 | 12,160 | 996 | 453 | 2,237,422 | 5.43 | 0.73 | 0.23 | 0.45 | 0.20 | 0.65 | -0.10 | -0.01 |  | 81.9 | 40.6 | 119.2 |
| 29 | 13,679 | 768 | 322 | 2,224,503 | 6.15 | 1.45 | 0.46 | 0.35 | 0.14 | 0.49 | -0.03 | 0.11 |  | 56.1 | 24.9 | 79.7 |
| 30 | 17,993 | 977 | 316 | 2,208,667 | 8.15 | 1.62 | 0.58 | 0.44 | 0.14 | 0.59 | 0.03 | -0.04 |  | 54.3 | 18.6 | 71.9 |
| 31 | 21,278 | 828 | 279 | 2,189,031 | 9.72 | 2.78 | 1.23 | 0.38 | 0.13 | 0.51 | -0.05 | 0.00 |  | 38.9 | 13.6 | 52.0 |
| 32 | 29,560 | 889 | 286 | 2,163,612 | 13.7 | 3.81 | 1.04 | 0.41 | 0.13 | 0.54 | 0.04 | 0.05 |  | 30.1 | 9.98 | 39.8 |
| 33 | 38,723 | 900 | 246 | 2,129,471 | 18.2 | 7.38 | 8.39 | 0.42 | 0.12 | 0.54 | -0.00 | -0.04 |  | 23.2 | 6.50 | 29.6 |
| 34 | 60,546 | 878 | 284 | 2,079,836 | 29.1 | 13.02 | -6.95 | 0.42 | 0.14 | 0.56 | 0.01 | 0.04 |  | 14.5 | 4.76 | 19.2 |
| 35 | 88,556 | 834 | 323 | 2,005,285 | 44.2 | 18.46 | 52.7 | 0.42 | 0.16 | 0.58 | 0.07 | 0.09 |  | 9.42 | 3.68 | 13.1 |
| 36 | 170,465 | 936 | 389 | 1,875,775 | 90.9 | 98.42 | 52.5 | 0.50 | 0.21 | 0.71 | 0.14 | -0.02 |  | 5.49 | 2.29 | 7.77 |
| 37 | 368,720 | 864 | 459 | 1,606,182 | 229.6 | 144.1 | 114.0 | 0.54 | 0.29 | 0.82 | 0.10 | 0.07 |  | 2.34 | 1.25 | 3.59 |
| 38 | 502,526 | 747 | 401 | 1,170,559 | 429.3 | 340.6 | 218.0 | 0.64 | 0.34 | 0.98 | 0.29 | 0.16 |  | 1.49 | 0.80 | 2.28 |
| 39 | 570,418 | 569 | 370 | 634,087 | 899.6 | 503.6 | 92.1 | 0.90 | 0.58 | 1.48 | 0.72 | 1.15 |  | 1.00 | 0.65 | 1.65 |
| 40 | 283,728 | 338 | 196 | 2,07,014 | 1,370.6 | 469.3 | -200.4 | 1.63 | 0.95 | 2.58 | 1.63 | -0.72 |  | 1.19 | 0.69 | 1.88 |
| 41 | 60,618 | 111 | 46 | 34,841 | 1,739.9 | 140.1 | -380.8 | 3.19 | 1.32 | 4.51 | 1.83 | 5.09 |  | 1.83 | 0.76 | 2.59 |
| 42 | 4,162 | 20 | 9 | 2,451 | 1,698.1 | 46.71 | 175.6 | 8.16 | 3.67 | 11.8 | 18.8 | 31.9 |  | 4.81 | 2.17 | 6.97 |
| 43 | 370 | 6 | 4 | 185 | 2,000.0 | 827.3 | 1740.7 | 32.4 | 21.6 | 54.1 | 71.62 | 76.8 |  | 16.2 | 10.99 | 27.0 |

* Derivatives of the birth rate (D1 and D2) estimated from splines fitted to the original rate (and not the observed rate).

Appendix Table S3. Numbers and Rates of Live births, Stillbirths, Neonatal Deaths and Perinatal Deaths Calculated Under the Fetuses-At-Risk and Births-

Based Formulations, **Twin Pregnancies**, United States 2004 to 2015.

| Gestational  age | Total | Still- | Neonatal | Fetuses-at-risk formulation | | | | | | | | |  | Births-based formulation | | |
| --- | --- | --- | --- | --- | --- | --- | --- | --- | --- | --- | --- | --- | --- | --- | --- | --- |
|  | births | births | deaths | Fetus- | Birth | Birth rate | | Still- | Neonatal | Perinatal | Perinatal death | |  | Still- | Neonatal | Perinatal |
|  |  |  |  | weeks | rate | derivatives* | | birth | death | death | rate derivatives | |  | birth | death | death |
|  |  |  |  | at risk |  | D1 | D2 | rate | rate | rate | D1 | D2 |  | rate | rate | rate |
| 20 | 6,826 | 3,654 | 2,866 | 1,582,595 | 4.31 | 0.08 | 2.06 | 2.31 | 1.81 | 4.12 | -0.31 | 3.30 |  | 535.3 | 903.5 | 955.2 |
| 21 | 7,583 | 3,541 | 3,683 | 1,575,391 | 4.81 | 0.76 | -0.25 | 2.25 | 2.34 | 4.59 | 0.86 | -0.48 |  | 467.0 | 911.2 | 952.7 |
| 22 | 8,638 | 3,119 | 4,824 | 1,567,280 | 5.51 | 0.47 | 0.12 | 1.99 | 3.08 | 5.07 | -0.27 | -1.30 |  | 361.1 | 874.1 | 919.5 |
| 23 | 9,336 | 1,805 | 4,600 | 1,558,293 | 5.99 | 0.88 | 0.11 | 1.16 | 2.95 | 4.11 | -1.18 | -0.46 |  | 193.3 | 610.8 | 686.1 |
| 24 | 10,871 | 1,267 | 3,371 | 1,548,190 | 7.02 | 0.55 | -0.33 | 0.82 | 2.18 | 3.00 | -1.21 | 0.33 |  | 116.5 | 351.0 | 426.6 |
| 25 | 11,101 | 888 | 1,978 | 1,537,204 | 7.22 | 0.6 | 0.39 | 0.58 | 1.29 | 1.86 | -0.72 | 0.53 |  | 80.0 | 193.7 | 258.2 |
| 26 | 12,895 | 901 | 1,430 | 1,525,206 | 8.45 | 1.35 | 1.18 | 0.59 | 0.94 | 1.53 | -0.32 | 0.21 |  | 69.9 | 119.2 | 180.8 |
| 27 | 15,067 | 718 | 977 | 1,511,225 | 9.97 | 2.23 | -0.2 | 0.48 | 0.65 | 1.12 | -0.22 | 0.10 |  | 47.7 | 68.1 | 112.5 |
| 28 | 18,668 | 760 | 857 | 1,494,357 | 12.5 | 1.82 | 1.02 | 0.51 | 0.57 | 1.08 | -0.12 | -0.02 |  | 40.7 | 47.9 | 86.6 |
| 29 | 21,256 | 617 | 585 | 1,474,395 | 14.4 | 3.81 | 0.87 | 0.42 | 0.40 | 0.82 | -0.21 | 0.00 |  | 29.0 | 28.3 | 56.5 |
| 30 | 29,054 | 560 | 513 | 1,449,240 | 20.0 | 5.59 | 6.8 | 0.39 | 0.35 | 0.74 | -0.05 | 0.24 |  | 19.3 | 18.0 | 36.9 |
| 31 | 39,351 | 585 | 427 | 1,415,038 | 27.8 | 14.0 | 2.55 | 0.41 | 0.30 | 0.72 | 0.10 | -0.01 |  | 14.9 | 11.0 | 25.7 |
| 32 | 62,710 | 719 | 461 | 1,364,007 | 46.0 | 16.1 | 14.6 | 0.53 | 0.34 | 0.87 | 0.02 | -0.01 |  | 11.5 | 7.44 | 18.8 |
| 33 | 86,437 | 637 | 420 | 1,289,434 | 67.0 | 39.2 | 14.5 | 0.49 | 0.33 | 0.82 | 0.13 | 0.13 |  | 7.37 | 4.90 | 12.2 |
| 34 | 14,4566 | 807 | 476 | 1,173,932 | 123.1 | 58.7 | 55.1 | 0.69 | 0.41 | 1.09 | 0.15 | -0.13 |  | 5.58 | 3.31 | 8.87 |
| 35 | 19,9090 | 665 | 424 | 1,002,104 | 198.7 | 120.9 | 10.4 | 0.66 | 0.42 | 1.09 | 0.09 | 0.26 |  | 3.34 | 2.14 | 5.47 |
| 36 | 28,3562 | 724 | 407 | 760,778 | 372.7 | 206.3 | 346.1 | 0.95 | 0.53 | 1.49 | 0.68 | 0.70 |  | 2.55 | 1.44 | 3.99 |
| 37 | 32,4277 | 624 | 454 | 456,859 | 709.8 | 587.2 | 4.07 | 1.37 | 0.99 | 2.36 | 1.01 | -0.29 |  | 1.92 | 1.40 | 3.32 |
| 38 | 23,1515 | 363 | 244 | 178,963 | 1,293.7 | 207.6 | -358.5 | 2.03 | 1.36 | 3.39 | 0.99 | 1.41 |  | 1.57 | 1.06 | 2.62 |
| 39 | 48,276 | 153 | 54 | 39,067 | 1,235.7 | 160.1 | 148.6 | 3.92 | 1.38 | 5.30 | 3.83 | 3.11 |  | 3.17 | 1.12 | 4.29 |
| 40 | 13,401 | 59 | 34 | 8,229 | 1,628.6 | 156.7 | -388.5 | 7.17 | 4.13 | 11.3 | 7.43 | 5.44 |  | 4.40 | 2.55 | 6.94 |
| 41 | 1,251 | 10 | 9 | 903 | 1,386.1 | -335.8 | -82.3 | 11.1 | 10.0 | 21.1 | 13.7 | 4.83 |  | 7.99 | 7.25 | 15.2 |
| 42 | 220 | 3 | 3 | 167 | 1,317.4 | 252.6 | 1,005.2 | 18.0 | 18.0 | 35.9 | 11.5 | -12.5 |  | 13.6 | 13.8 | 27.3 |
| 43 | 57 | 0 | 1 | 29 | 2,000.0 | 1,167.1 | 570.2 | 0.0 | 35.1 | 35.1 | -17.7 | -49.2 |  | 0.00 | 17.5 | 17.5 |

* Derivatives of the birth rate (D1 and D2) estimated from splines fitted to the original rate (and not the observed rate).

The perinatal death rate among twins was 33.3 per 1,000 total births.

Appendix Table S4. Numbers and Rates of Live births, Stillbirths, Neonatal Deaths and Perinatal Deaths Calculated Under the Fetuses-At-Risk and Birt

Based Formulations, **Triplet Pregnancies**, United States 2004 to 2015.

| Gestational  age | Total | Still- | Neonatal | Fetuses-at-risk formulation | | | | | | | | |  | Births-based formulation | | |
| --- | --- | --- | --- | --- | --- | --- | --- | --- | --- | --- | --- | --- | --- | --- | --- | --- |
|  | births | births | deaths | Fetus- | Birth | Birth rate | | Still- | Neonatal | Perinatal | Perinatal death | |  | Still- | Neonatal | Perinatal |
|  |  |  |  | weeks | rate | derivatives* | | birth | death | death | rate derivatives | |  | birth | death | death |
|  |  |  |  | at risk |  | D1 | D2 | rate | rate | rate | D1 | D2 |  | rate | rate | rate |
| 20 | 703 | 293 | 371 | 63,170 | 11.1 | -0.78 | 13.1 | 4.64 | 5.87 | 10.5 | -0.38 | 8.29 |  | 416.8 | 904.9 | 944.5 |
| 21 | 785 | 315 | 427 | 62,426 | 12.6 | 2.64 | -2.97 | 5.05 | 6.84 | 11.9 | 2.16 | -1.79 |  | 401.3 | 908.5 | 945.2 |
| 22 | 883 | 288 | 506 | 61,592 | 14.3 | -0.15 | 0.66 | 4.68 | 8.22 | 12.9 | -1.11 | -3.33 |  | 326.2 | 850.4 | 899.2 |
| 23 | 876 | 155 | 457 | 60,712 | 14.4 | 3.53 | 3.00 | 2.55 | 7.53 | 10.1 | -3.12 | -0.72 |  | 176.9 | 633.8 | 698.6 |
| 24 | 1,202 | 69 | 371 | 59,673 | 20.1 | 3.44 | -1.88 | 1.16 | 6.22 | 7.37 | -2.98 | 0.63 |  | 57.4 | 327.4 | 366.1 |
| 25 | 1,222 | 58 | 199 | 58,461 | 20.9 | 2.12 | 0.30 | 0.99 | 3.40 | 4.40 | -2.03 | 1.47 |  | 47.5 | 171.0 | 210.3 |
| 26 | 1,494 | 52 | 148 | 57,103 | 26.2 | 6.13 | 8.76 | 0.91 | 2.59 | 3.50 | -0.52 | 0.88 |  | 34.8 | 102.6 | 133.9 |
| 27 | 1,898 | 70 | 89 | 55,407 | 34.3 | 13.4 | -1.46 | 1.26 | 1.61 | 2.87 | -0.48 | -0.34 |  | 36.9 | 48.7 | 83.8 |
| 28 | 2,619 | 73 | 60 | 53,149 | 49.3 | 9.54 | 7.29 | 1.37 | 1.13 | 2.50 | -0.57 | 0.32 |  | 27.9 | 23.6 | 50.8 |
| 29 | 2,,997 | 50 | 46 | 50,341 | 59.5 | 24.3 | 4.86 | 0.99 | 0.91 | 1.91 | -0.13 | 0.10 |  | 16.7 | 15.6 | 32.0 |
| 30 | 4,421 | 50 | 45 | 46,632 | 94.8 | 30.0 | 34.6 | 1.07 | 0.97 | 2.04 | -0.31 | 0.04 |  | 11.3 | 10.3 | 21.5 |
| 31 | 5,557 | 35 | 28 | 41,643 | 133.4 | 77.6 | 16.9 | 0.84 | 0.67 | 1.51 | 0.20 | 0.72 |  | 6.30 | 5.07 | 11.3 |
| 32 | 8,165 | 58 | 27 | 34,782 | 234.8 | 79.5 | 46.4 | 1.67 | 0.78 | 2.44 | 0.74 | 0.22 |  | 7.10 | 3.33 | 10.4 |
| 33 | 8,618 | 49 | 26 | 26,390 | 326.6 | 183.9 | 116.6 | 1.86 | 0.99 | 2.84 | 0.85 | 0.34 |  | 5.69 | 3.03 | 8.70 |
| 34 | 10,241 | 46 | 24 | 16,961 | 603.8 | 292.1 | 125.2 | 2.71 | 1.42 | 4.13 | 0.93 | -1.00 |  | 4.49 | 2.35 | 6.84 |
| 35 | 7,306 | 30 | 12 | 8,187 | 892.4 | 345.3 | -133.1 | 3.66 | 1.47 | 5.13 | 2.29 | 7.98 |  | 4.11 | 1.65 | 5.75 |
| 36 | 3,331 | 25 | 4 | 2,869 | 1,161.2 | -1.06 | -472.2 | 8.72 | 1.39 | 10.1 | 7.86 | -10.1 |  | 7.51 | 1.21 | 8.71 |
| 37 | 726 | 12 | 0 | 840 | 864.3 | -425.4 | -290.4 | 14.3 | 0.00 | 14.3 | -6.24 | 6.99 |  | 16.5 | 0.00 | 16.5 |
| 38 | 205 | 6 | 0 | 375 | 547.4 | -138.9 | 1,219.9 | 16.0 | 0.00 | 16.0 | 34.9 | 63.2 |  | 29.3 | 0.00 | 29.3 |
| 39 | 164 | 13 | 3 | 190 | 863.2 | 977.8 | -380.0 | 68.4 | 15.8 | 84.2 | 76.6 | -11.2 |  | 79.3 | 19.9 | 97.6 |
| 40 | 96 | 7 | 1 | 60 | 1,600.0 | -614.3 | -1,125.9 | 116.7 | 16.7 | 133.3 | 10.7 | -91.0 |  | 72.9 | 11.2 | 83.3 |
| 41 | 4 | 1 | 0 | 10 | 400.0 | 89.8 | 2,219.6 | 100.0 | 0.00 | 100.0 | -72.0 | -70.5 |  | 250.0 | 0.00 | 250.0 |
| 42 | 8 | 0 | 0 | 4 | 2,000.0 | 1,455.1 | -1,544.5 | 0.00 | 0.00 | 0.00 | -122.6 | -26.9 |  | 0.00 | 0.00 | 0.00 |
| 43 | 0 | 0 | 0 | 0 |  | -7110.2 | -17,641.7 |  |  |  |  |  |  |  |  |  |

* Derivatives of the birth rate (D1 and D2) estimated from splines fitted to the original rate (and not the observed rate).

The perinatal death rate among triplets was 72.4 per 1,000 total births.
