# Supplementary figures and images for: Towards a unified perinatal theory: Reconciling the births‐based and fetus‐at‐risk models of perinatal mortality
Source: Paediatr Perinat Epidemiol. 2019 Jan 22;33(2):101–12. doi: 10.1111/ppe.12537 (PMC6487839; doi:10.1111/ppe.12537)

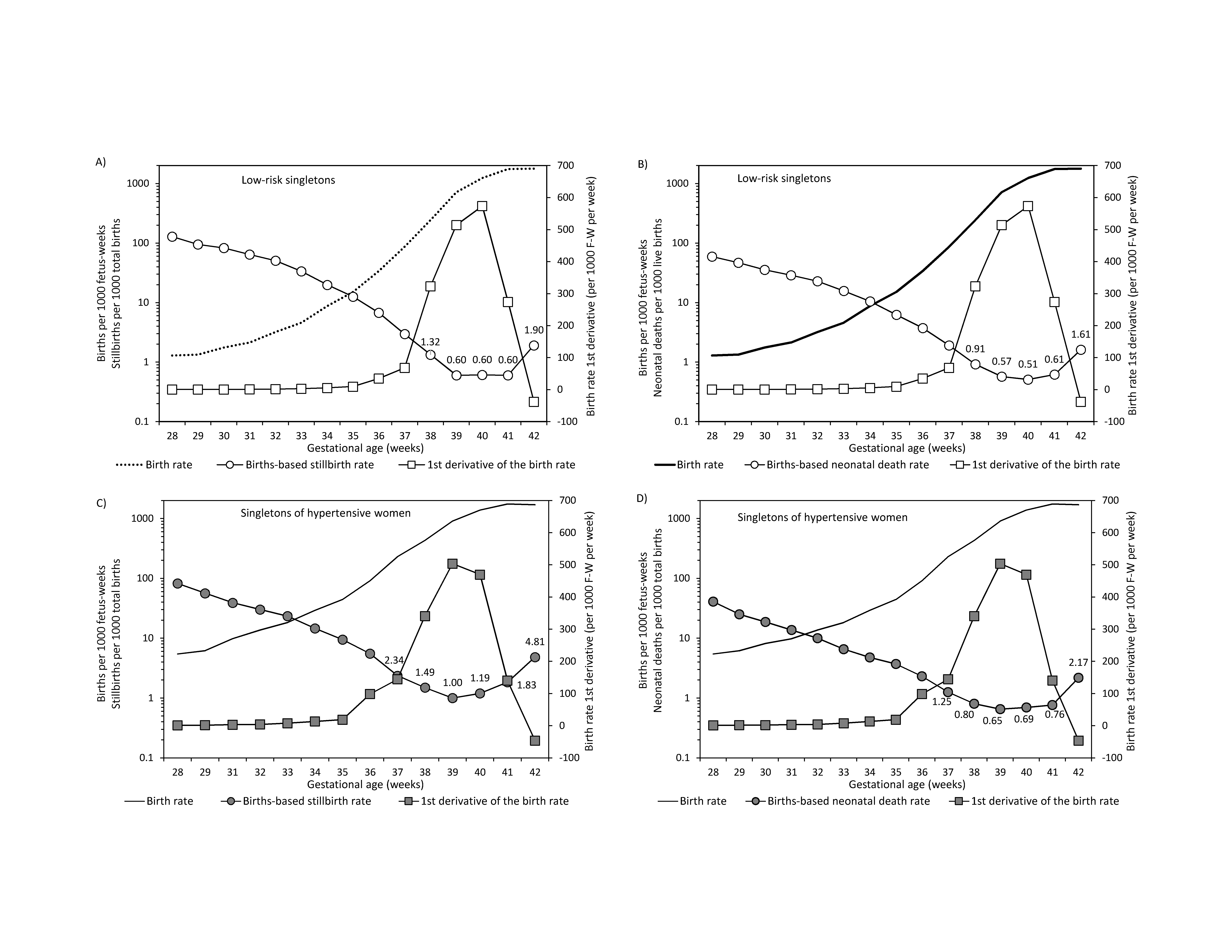

Supplement: Supplementary file 2 [file PPE-33-101-s002.jpg]

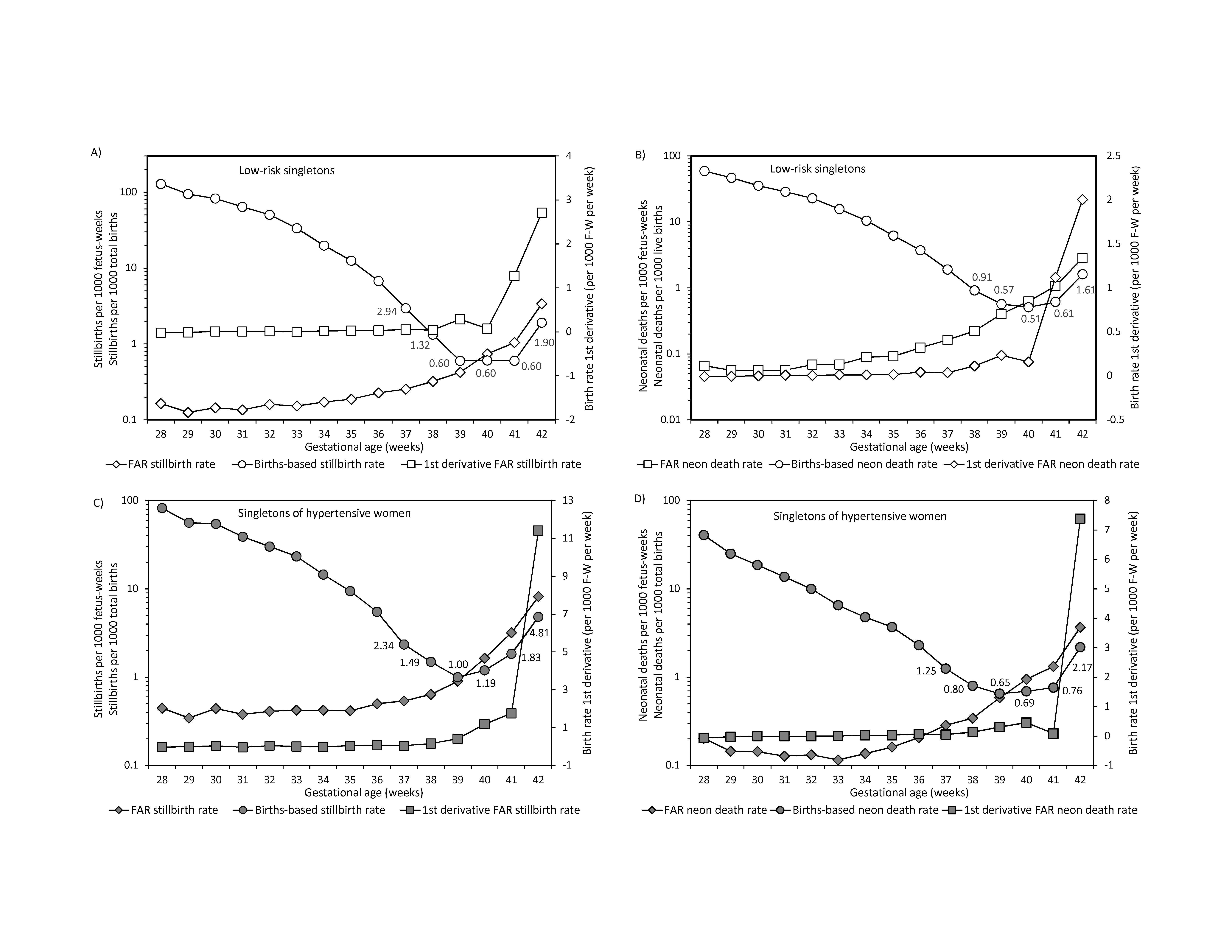

Supplement: Supplementary file 3 [file PPE-33-101-s003.jpg]

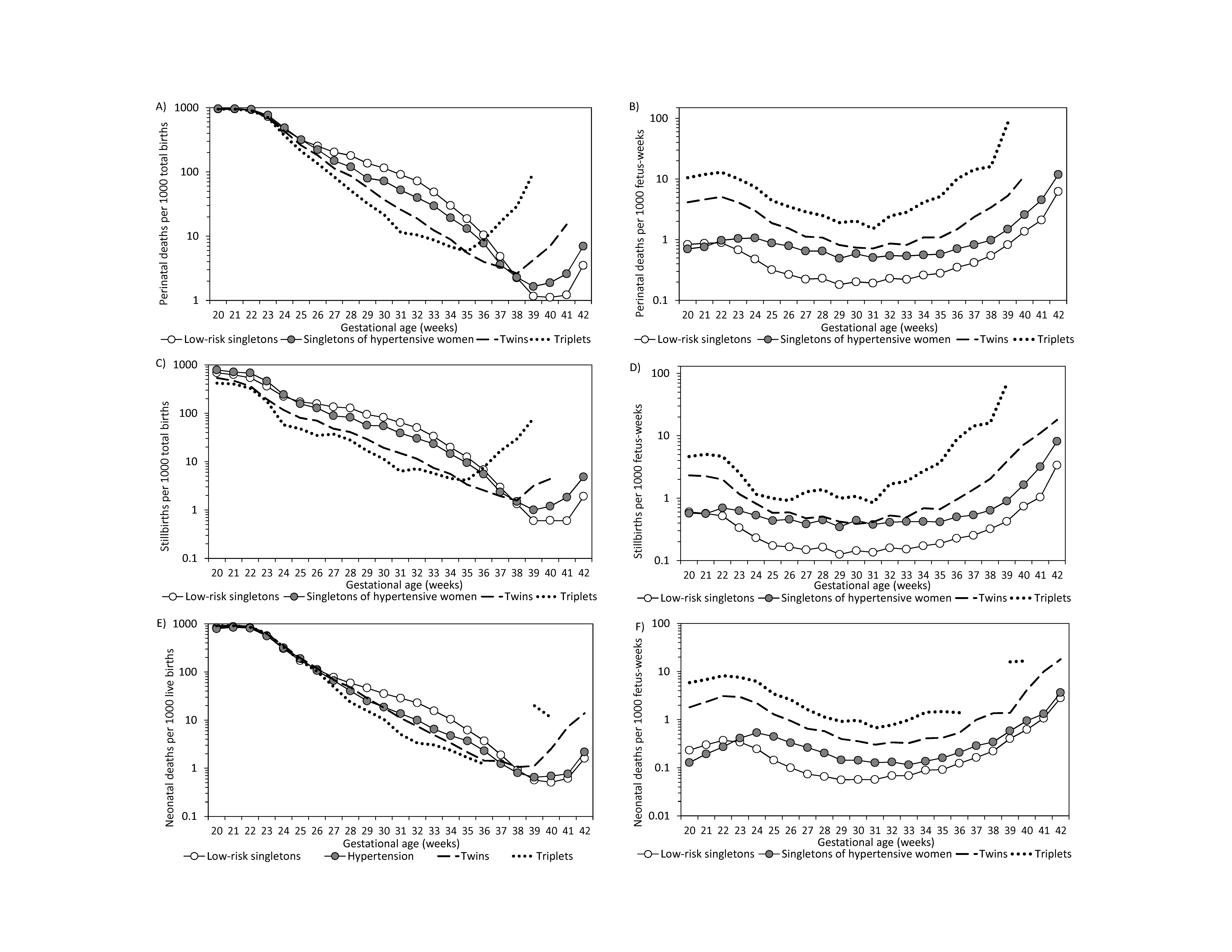

Supplement: Supplementary file 4 [file PPE-33-101-s004.jpg]

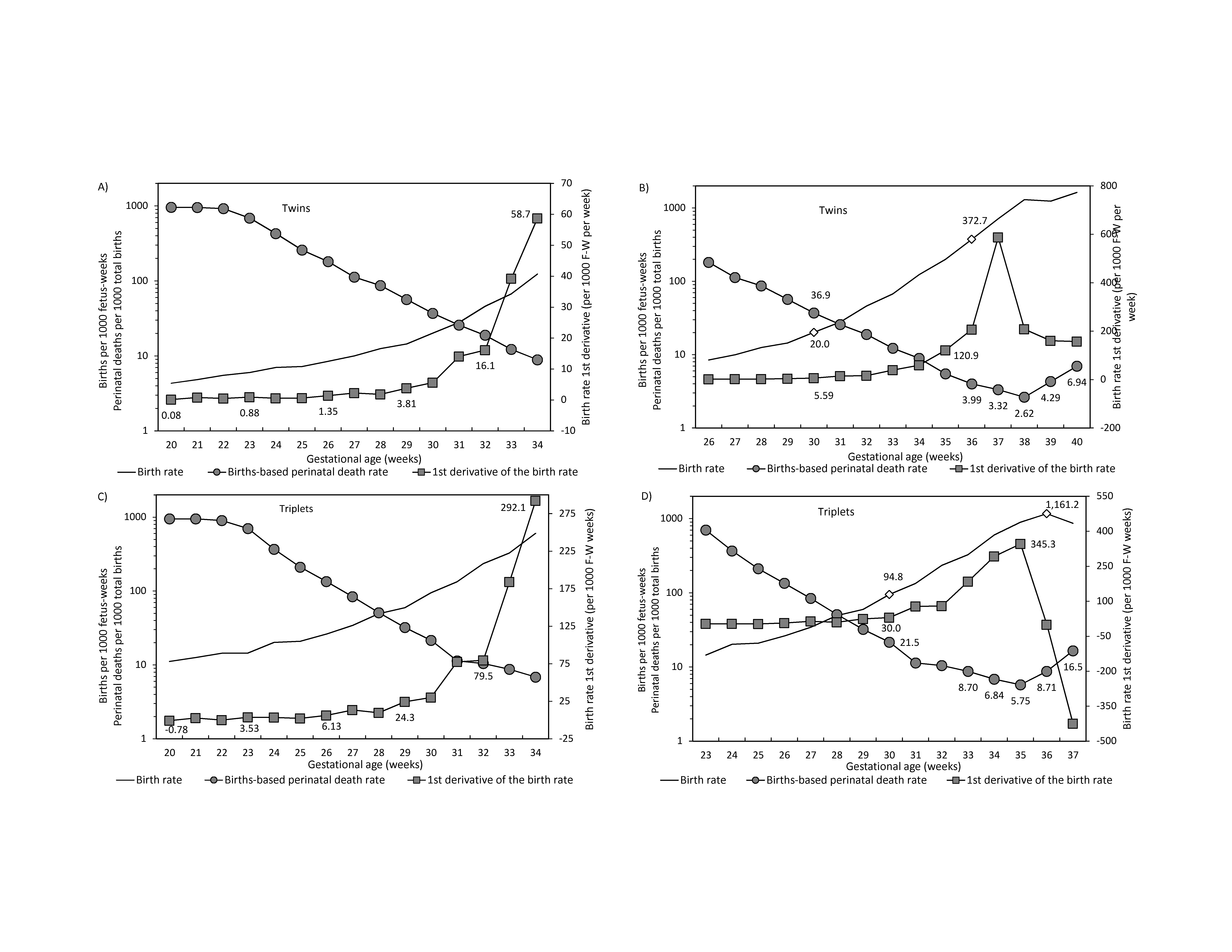

Supplement: Supplementary file 5 [file PPE-33-101-s005.jpg]

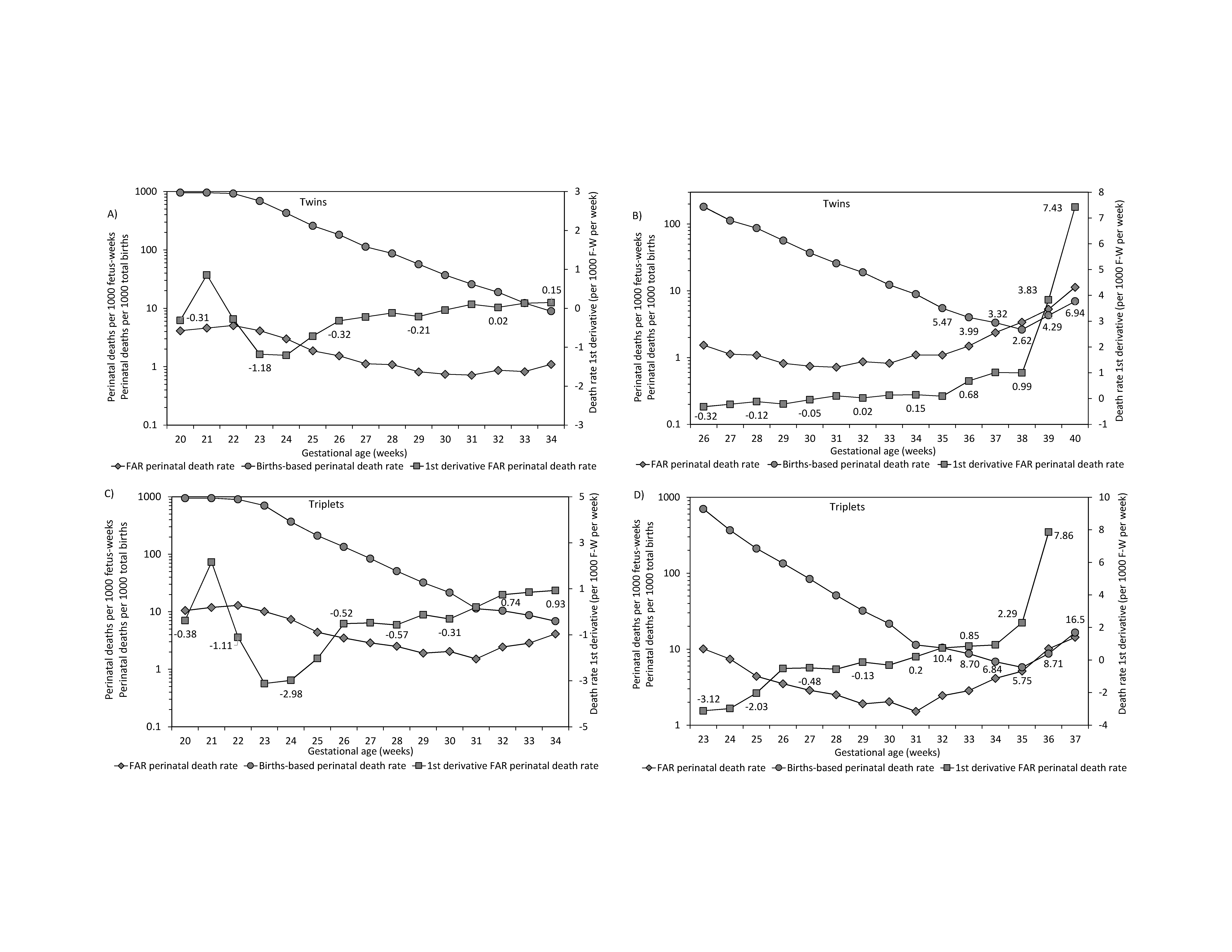

Supplement: Supplementary file 6 [file PPE-33-101-s006.jpg]

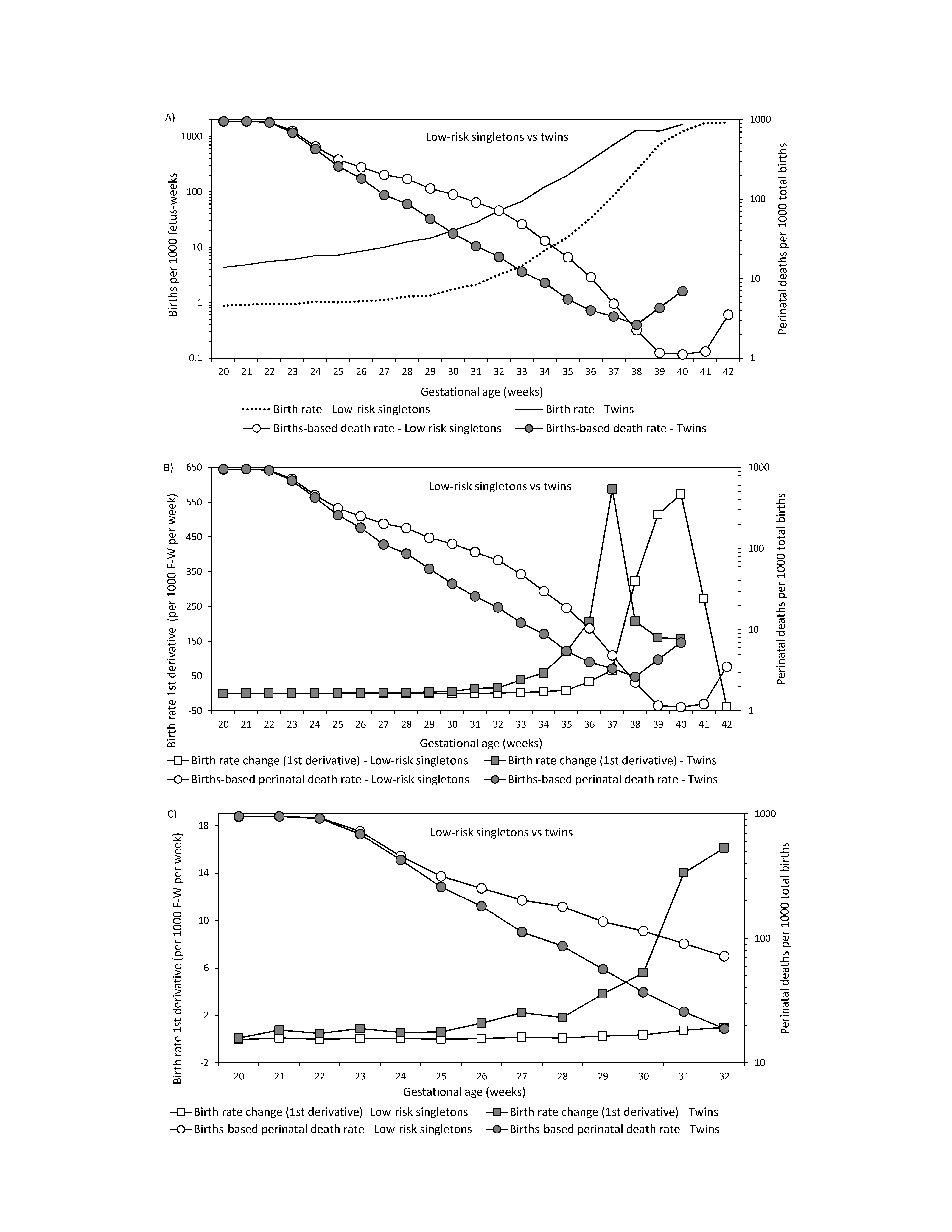

Supplement: Supplementary file 7 [file PPE-33-101-s007.jpg]

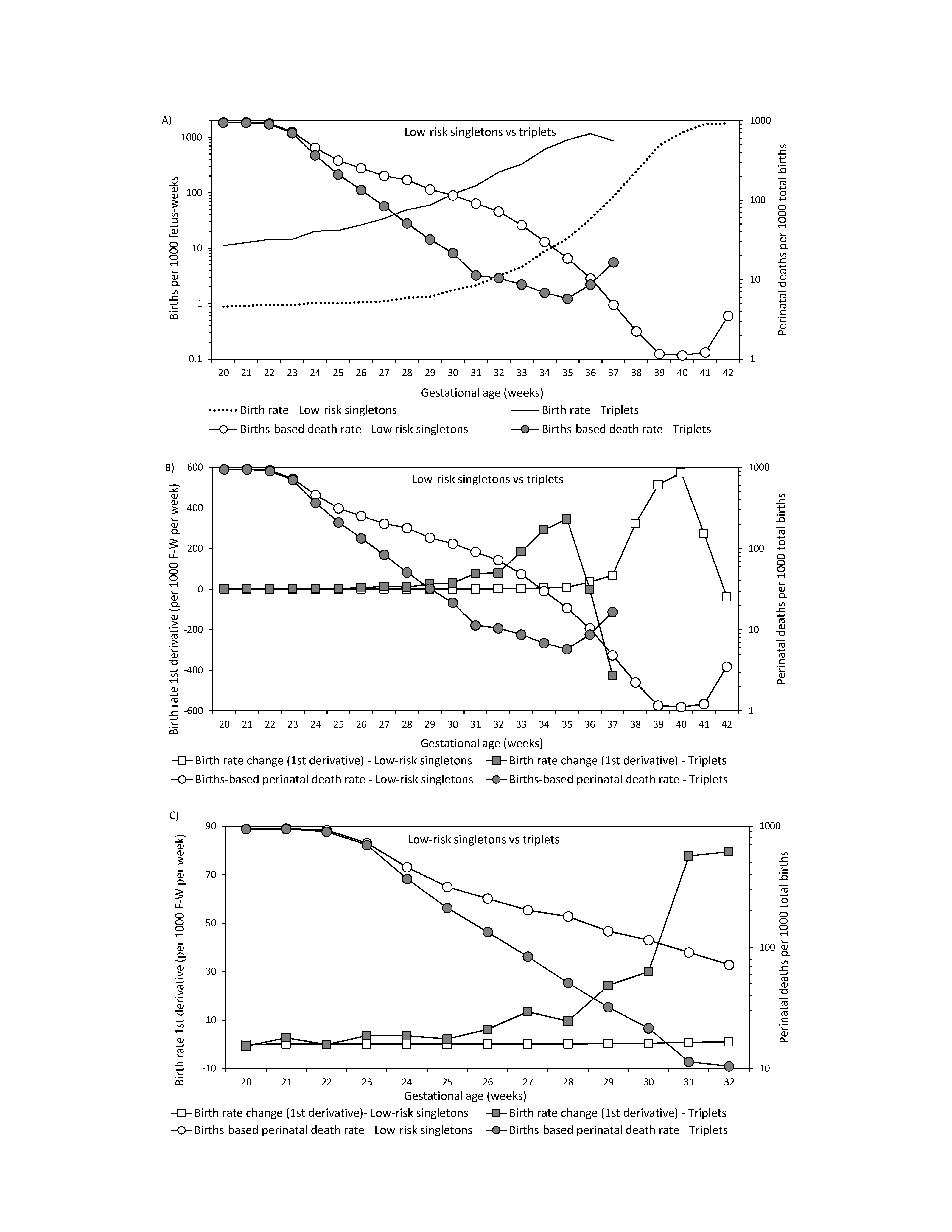

Supplement: Supplementary file 8 [file PPE-33-101-s008.jpg]
